# The effect of intraoperative goal-directed fluid therapy combined with enhanced recovery after surgery program on postoperative complications in elderly patients undergoing thoracoscopic pulmonary resection: a prospective randomized controlled study

**DOI:** 10.1186/s13741-023-00327-x

**Published:** 2023-07-10

**Authors:** Hongmei Ma, Xin Li, Zhe Wang, Qiao Qiao, Yanfeng Gao, Hui Yuan, Bin Guan, Zheng Guan

**Affiliations:** 1 Department of Anesthesiology, the First Affiliated Hospital of Xi’an Jiaotong University, Xi’an, Shaanxi China; 2 Department of Anesthesiology, Qinghai Provincial People’s Hospital, Xining, Qinghai China; 3 Department of Thoracic Surgery, the First Affiliated Hospital of Xi’an Jiaotong University, Xi’an, Shaanxi China

**Keywords:** Goal-directed fluid therapy, Enhanced recovery after surgery, Acute kidney injury, Pulmonary resection

## Abstract

**Background:**

To investigate the effect of intraoperative goal-directed fluid therapy (GDFT) combined with enhanced recovery after surgery (ERAS) program on postoperative complications in elderly patients undergoing thoracoscopic pulmonary resection.

**Methods:**

Patients, more than 60 years old, undergoing thoracoscopic pulmonary resection for non-small cell lung cancer were randomly divided into GDFT group and restrictive fluid therapy (RFT) group. ERAS program was implemented in all patients. In GDFT group, the intraoperative fluid management was guided by stroke volume variation (SVV), cardiac index (CI), and mean arterial pressure (MAP) and maintained the *SVV* < 13%, *CI* > 2.5 L/min/m^2^, and *MAP* > 65 mmHg. In RFT group, fluid maintenance with 2 ml/kg/h of balanced crystalloid solution, norepinephrine was used to maintain *MAP* > 65 mmHg. The incidence of postoperative acute kidney injury (AKI) and pulmonary and cardiac complications was compared.

**Results:**

Two-hundred seventy-six patients were enrolled and randomly divided into two groups (138 in each group). Compared to RFT group, the total intraoperative infusion volume, colloids infusion volume, and urine output were more; the dosage of norepinephrine was lower in GDFT group. Although there were no significant differences of postoperative AKI (GDFT vs RFT; 4.3% vs 8%; *P* = 0.317) and composite postoperative complications (GDFT vs RFT; 66 vs 70) between groups, but the postoperative increase degree of serum creatinine was lower in GDFT group than that in RFT group (GDFT vs RFT; 91.9 ± 25.2 μmol/L vs 97.1 ± 17.6 μmol/L; *P* = 0.048).

**Conclusions:**

Under ERAS program, there was no significant difference of AKI incidence between GDFT and RFT in elderly patients undergoing thoracoscopic pulmonary resection. But postoperative increase degree of serum creatinine was lower in GDFT group.

**Trial registration:**

Registered at ClinicalTrials.gov, NCT04302467 on 26 February 2020.

## Background

Lung cancer is the most common cause of cancer-related death worldwide. According to the WHO, in 2020, more than 2 million new lung cancer cases were reported worldwide, and there were 1.8 million deaths (World Health Organization 2020). Thoracoscopic pulmonary resection is the recommended procedure for lung cancer treatment (Howington et al. 2013); it is associated with a high incidence of postoperative acute kidney injury (AKI) (Romagnoli and Ricci 2015), ranging from 1.8 to 14.2% (Wu et al. 2019; Naruka et al. 2019). Postoperative AKI could increase reintubation rate and prolong mechanical ventilation and hospitalization length of stay (LOS) after lung surgery (Ishikawa et al. 2012).

Enhanced recovery after surgery (ERAS) integrates perioperative interventions to maintain physiological function, decrease postoperative complications, and facilitate postoperative recovery. In thoracic and lung surgery, ERAS program could reduce intensive care unit (ICU) admission; shorten thoracic drainage duration, ICU, and hospitalization LOS (Peng et al. 2021; Abrão et al. 2021); and decrease postoperative pulmonary and cardiovascular complications (Forster et al. 2021; Wang et al. 2021; Li et al. 2021). Perioperative fluid management is one key component of ERAS program; though intraoperative fluid therapy should aim to maintain euvolemia with an individualized approach, a restrictive, zero-balance approach to intraoperative fluid management may also be reasonable in some ERAS program (Zhu et al. 2019), such as the ERAS program in lung surgery (Batchelor et al. 2019). Restrictive fluid therapy (RFT) may induce hypotension and organ hypoperfusion; they are important risk factors of postoperative AKI. Both in colorectal cancer resection surgery (Shim et al. 2020) and radical cystectomy surgery (Hanna et al. 2020), ERAS program with zero-balance fluid therapy or RFT was associated with higher incidence of AKI. So, AKI should be concerned when RFT was implemented in ERAS program.

Goal-directed fluid therapy (GDFT) used either fluids or in combination with inotropes and vasopressors to achieve specific hemodynamic parameters (Kaufmann et al. 2019). Studies showed that GDFT significantly reduced postoperative AKI in high-risk patients (Giglio et al. 2019) and in low-moderate risk patients undergoing moderate risk surgery (Vecino et al. 2018). The American Society for Enhanced Recovery recommended to adopt GDFT in high-risk patients or in high-risk procedures (Makaryus et al. 2018). Perioperative Anesthesia Care in Thoracic Surgery group in Italian also recommended GDFT in high-risk patients undergoing thoracic surgery (Piccioni et al. 2020). However, the benefit of GDFT often seen in standard care pathways may be masked by ERAS program which can also maintain optimal physiological function. There was no significant difference of AKI incidence after colorectal (Vaca et al. 2021) and thoracoscopic lobectomy surgery (Li and Peng 2021), as well as serum creatinine level after colectomy surgery (Srinivasa et al. 2013) between GDFT and RFT combined with ERAS program. High-risk patients are more likely to experience postoperative complications and may benefit more from GDFT. It is still unclear whether GDFT combined with ERAS program could reduce the incidence of AKI in high-risk patients undergoing thoracoscopic pulmonary resection.

Age was a risk factor of postoperative AKI (Löffel et al. 2020). One study showed that in patients 60 years or older, there was a nearly 2-fold increased risk of developing AKI compared to that in younger patients after thoracic surgery (Naruka et al. 2019). In this study, we conducted a prospective randomized controlled trial to compare the effect of GDFT and RFT combined with ERAS program on the incidence of postoperative AKI in elderly patients undergoing thoracoscopic pulmonary resection. The postoperative cardiopulmonary complications were also investigated.

## Methods

### Ethics

This prospective, randomized, double-blind (patients and outcome assessors) trial was approved by Ethics Committee of the First Affiliated Hospital of Xi’an Jiaotong University, Xi’an, China (ref.: XJTU1AF2019LSL-012), on 3 December, 2019, and registered at the ClinicalTrials.gov (ref.: NCT04302467) before the first patient was enrolled. This study was conducted in a tertiary teaching hospital in Shaanxi, People’s Republic of China, and followed the guidelines of the CONSORT criteria. There were slight changes from the primary protocol. The indicators reflecting lung function were removed from inclusion criteria, because they were the risk factors of postoperative pulmonary complications (PPCs), which was not the primary aim of our study. The expected operation duration < 2 h was added in exclusion criteria, because longer anesthesia and operation time were risk factors of AKI. The incidence of infection and cardiac infarction was removed from secondary aims, because the infection complications had high heterogeneity with other complications; it also has little relationship with our interventions. The incidence of cardiac infarction was low, it is often based on the incidence within 30 days after surgery, and our follow-up only lasted until the patient was discharged.

### Inclusion and exclusion criteria

The patient enrollment was conducted from May 2020 to June 2022. Patients, more than 60 years old, undergoing thoracoscopic pulmonary resection for non-small cell lung cancer, were enrolled in the study after written informed consent was signed. Exclusion criteria included preoperative serum creatinine > 176 μmol/L; blood urea nitrogen (BUN) > 7.1 mmol/L; N-terminal pro-B-type natriuretic peptide (NT-proBNP) > 300 pg/ml; albumin < 30 g/L; hemoglobin < 100 g/L, known allergy to hydroxyethyl starch (HES); and the expected operation duration < 2 h.

### Classification and randomization

**Fig. 1:**
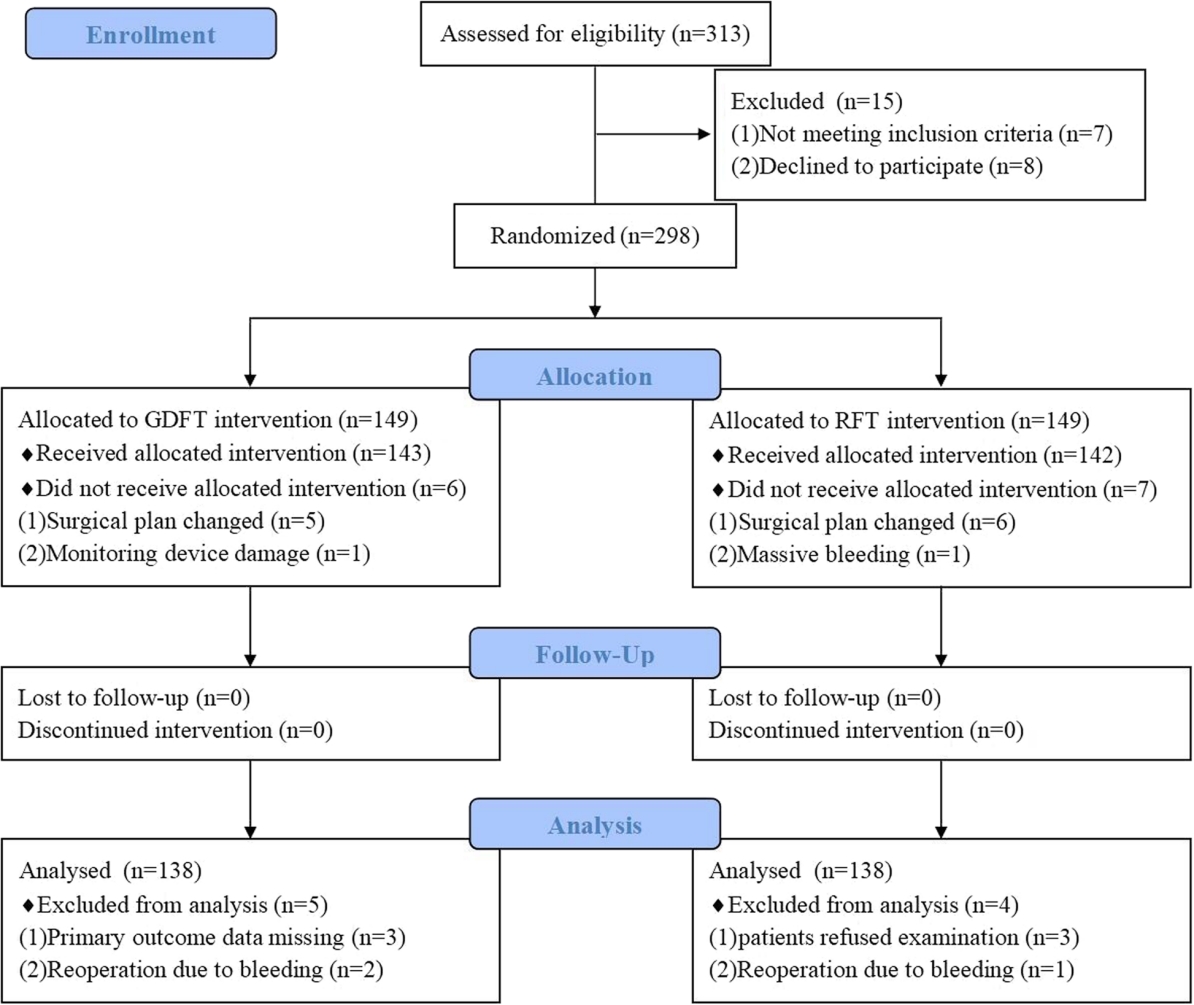
CONSORT flow diagram. *GDFT* goal-directed fluid therapy, *RFT* restrictive fluid therapy

Patients were randomly divided into GDFT group and RFT group at a 1:1 ratio using random numbers generated by Microsoft Excel (Fig. 1). The randomization codes were kept in sealed envelopes marked by serial numbers and opened by anesthesiologists once patients entered the operation room. The randomization was performed by an assistant who was not involved in this study; the information was blinded to the patients and outcome assessors.

### ERAS program

We established an ERAS program for patients undergoing thoracoscopic pulmonary resection, including preoperative respiratory function exercise, nutritional optimization, smoking cessation for the smokers, fasting for 8 h before operation, oral intake 300 ml of carbohydrate 3 h prior to operation, total intravenous anesthesia using short-acting drugs without premedication, one-lung ventilation (OLV) using double-lumen tube intubation, and lung-protective ventilation strategy. Thoracoscopic pulmonary resection was performed using two- to three-hole techniques. Postoperative analgesia is using paravertebral nerve block and/or patient-controlled intravenous analgesia. Urinary catheter was removed immediately after anesthesia recovery, thoracic drainage tube was removed if drainage volume was less than 5 ml/kg/day, resumption of oral fluid intake 4–6 h after operation if there was no nausea and vomiting, meanwhile parenteral fluids discontinued, and early mobilization after operation was encouraged.

### Intraoperative hemodynamic monitoring and fluid therapy protocol

Invasive arterial blood pressure was monitored through radial artery catheter in all the patients. Stroke volume variation (SVV) and cardiac index (CI) were obtained through FloTrac/Vigileo sensor (Edwards Lifesciences, Irvine, CA, USA) by analysis of the arterial curve. In RFT group, the SVV and CI were also obtained, but the information was hidden to anesthesiologists.

**Fig. 2:**
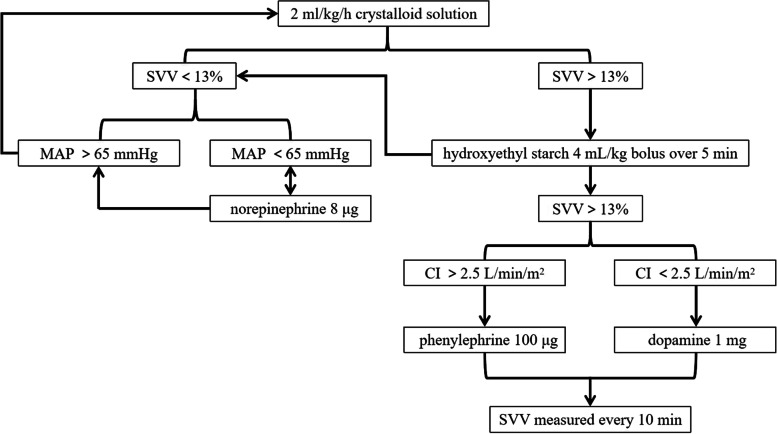
The GDFT protocol. *SVV* stroke volume variation, *MAP* mean arterial pressure, *CI* cardiac index

In GDFT group, fluid maintenance is with 2 ml/kg/h of balanced crystalloid solution. When *SVV* > 13%, 4 mL/kg HES was bolus infused within 5 min; if SVV was still more than 13%, 100 μg of phenylephrine was injected when *CI* > 2.5 L/min/m^2^, or 1 mg of dopamine was injected when *CI* < 2.5 L/min/m^2^. When *SVV* < 13%, but mean arterial pressure (MAP) < 65 mmHg, 8 μg of norepinephrine was injected. The hemodynamic parameters were evaluated every 10 min. The GDFT protocol was shown in Fig. 2.

In RFT group, fluid maintenance with 2 ml/kg/h of balanced crystalloid solution, blood loss was compensated by infusion of HES in a 1:1 ratio. Norepinephrine was used to maintain MAP above 65 mmHg.

### Primary and secondary aims and outcome measurement

The primary aim was to see if ERAS program combined with GDFT can reduce the incidence of AKI 48 h after operation. The secondary aims were to compare the incidence of acute lung injury (ALI) 24 h after operation, heart failure 48 h after operation, myocardia injury after noncardiac surgery (MINS) 7 days after operation, pneumonia and new onset arrhythmia during hospitalization, ICU, and hospitalization LOS.

AKI is defined as 50% relative increase or 26.5 μmol/L absolute increase of creatinine from the baseline. ALI is defined as oxygen index (oxygen partial pressure/fraction of inhaled oxygen) < 300 mmHg, bilateral lung infiltration in chest x-ray, excluding cardiogenic pulmonary edema. Heart failure is defined as NT-proBNP increased above 900 pg/ml. MINS is defined as troponin T increased above 0.03 ng/ml and/or creatine kinase-MB (CK-MB) increased above 8.8 ng/ml.

### Sample size calculation and statistical analysis

The primary aim is the incidence of AKI 48 h after operation. Previous study showed that the incidence of AKI was 3.6% in liberal infusion group and 1.2% in GDFT group after lung surgery (Xu et al. 2017). GDFT and RFT with ERAS program all can reduce the incidence of AKI compared to traditional care, including liberal fluid therapy; we hypothesized that the intervention efficacy of GDFT and RFT was consistent and used the data of AKI incidence between liberal fluids management and GDFT to calculate the sample size to achieve a statistical power of 0.8 and alpha error of 0.05 using two-sided non-inferiority test; accounting for 10% dropouts, 138 patients were required in each group.

The statistical analysis was performed using SPSS 26.0 software. The data assessment and analysis were performed by an independent research staff supervised by an independent statistician. Data were assessed for normality using the Kolmogorov–Smirnov test. The normally distributed data were presented as mean ± SD and compared using the independent *t*-test between groups and paired *t*-test within groups. The nonnormally distributed data were presented as median [interquartile range (IQR)] and compared using the Mann–Whitney *U*-test for unpaired data and Wilcoxon rank-sum test for paired data, respectively. The categorical data were presented as number (percentage) and compared using the chi-square test. *P*-value less than 0.05 was considered to be statistical significance.

## Results

### Patient’s demographics and baseline characteristics

From May 2020 to June 2022, 298 patients were enrolled in our study; 138 patients were final analyzed in each group. There were no significant differences in terms of demographics, comorbidities, pulmonary function, renal function, myocardial enzyme levels, and tumor stage between the two groups (Table 1).

**Table 1:** Patients demographics and baseline characteristics

Parameters	GDFT group (*N* = 138)	RFT group (*N* = 138)	*p*-value
Age (years)	66.3 ± 4.6	66.7 ± 3.2	0.379
Male sex [*n* (%)]	88 (63.8%)	76 (55.1%)	0.177
BMI (kg/m^2^)	23.1 ± 3.2	23.3 ± 3.2	0.610
ASA classification			0.723
I level [*n* (%)]	18 (13.0%)	16 (11.6%)	
II level [*n* (%)]	95 (68.8%)	101 (73.2%)	
III level [*n* (%)]	25 (18.2%)	21 (15.2%)	
Comorbidities			
Hypertension [*n* (%)]	51 (37.0%)	62 (44.9%)	0.221
Diabetes mellitus [*n* (%)]	34 (24.6%)	29 (21.0%)	0.321
Coronary artery disease [*n* (%)]	26 (18.8%)	31 (22.5%)	0.552
COPD [*n* (%)]	48 (34.8%)	57 (41.3%)	0.697
Smoking			
History [*n* (%)]	117 (85%)	112 (81%)	0.522
Smoking index (*n*/d × years)	340 (240–580)	360 (278–558)	0.225
Preoperative hemoglobin (g/l)	123.8 ± 12.7	121.4 ± 12.4	0.116
Preoperative *FEV*_1_ (%)	2.7 ± 0.7	2.6 ± 0.6	0.993
Oxygenation index (mmHg)	384 (356–436)	381 (341–436)	0.470
Preoperative BUN (mmol/l)	4.1 ± 1.5	4.2 ± 1.4	0.895
Preoperative creatinine (μmol/l)	90.0 ± 27.8	86.5 ± 18.3	0.217
Preoperative NT-proBNP (pg/ml)	171.8 ± 54.9	166.6 ± 48.4	0.407
Preoperative CK-MB (ng/ml)	3.5 ± 1.3	3.3 ± 1.3	0.201
Preoperative troponin T (ng/ml)	0.009 (0.007–0.011)	0.009 (0.007–0.012)	0.586
Tumor stage			0.620
Tis	25	24	
TI	15	18	
TII	76	81	
TIII	22	15	

Continuous data are presented as mean ± SD or median [interquartile range (IQR)]; categorical data are presented as number (percentage)

*Abbreviations*: *BMI* Body mass index, *ASA* American society of anesthesiologists, *COPD* Chronic obstructive pulmonary disease, *FEV*_1_ Forced expiratory volume in 1 s, *BUN* Blood urea nitrogen, *NT-proBNP* N-terminal pro-B-type natriuretic peptide, *CK-MB* Creatine kinase-MB

### ERAS program implementation rate

**Fig. 3:**
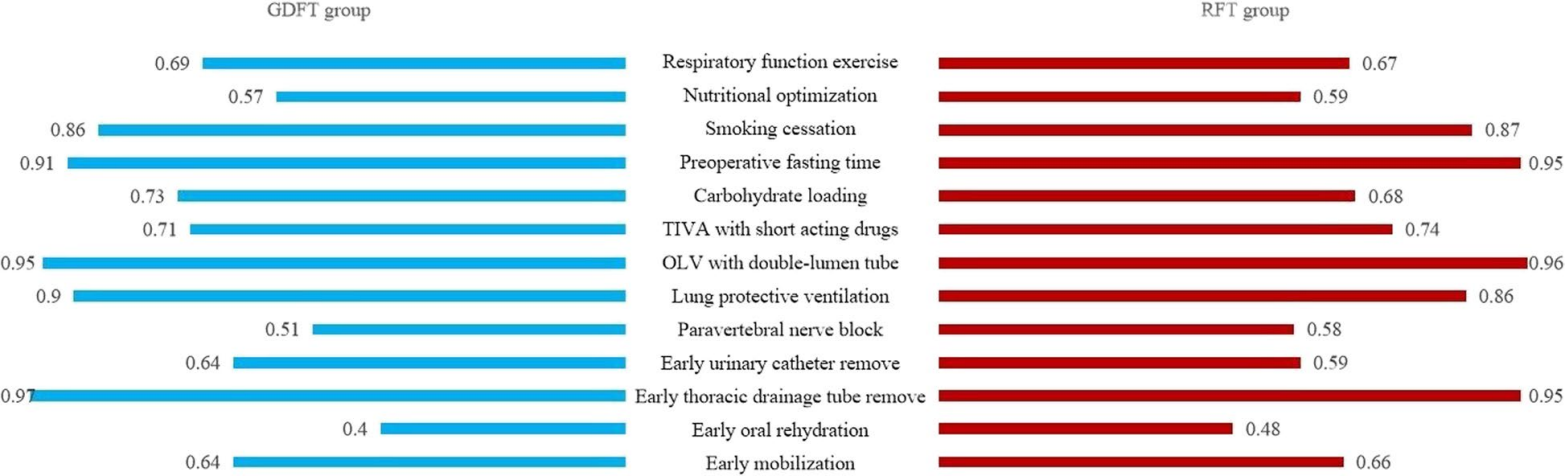
The ERAS program implementation rate. *TIVA* total intravenous anesthesia, *OLV* one-lung ventilation

There were 13 items in our ERAS program. The ERAS program implementation rate was defined as the number of patients who received items divided by the total number of patients. There had high implementation rate in items such as anesthesia management, preoperative fasting time, and early thoracic drainage tube remove. However, there also had low implementation rate in items such as nutritional optimization, paravertebral nerve block, and early oral rehydration. There were no significant differences of ERAS program implementation rate between the two groups (Fig. 3).

### Perioperative characteristics

There were no significant differences in the surgery and anesthesia characteristics between the two groups. In the crystalloid infusion volume and blood product transfusion volume, estimated blood loss was similar between the two groups. The total infusion volume, colloid infusion volume, and urine output were more in GDFT group than that in RFT group. Dopamine and phenylephrine were only used in GDFT group; the dosage of norepinephrine was lower in GDFT group than that in RFT group. There were no significant differences in first mobilization time, ICU, and hospitalization LOS between the two groups (Table 2).

**Table 2:** Perioperative characteristics

Parameters	GDFT group (*N* = 138)	RFT group (*N* = 138)	*p*-value
Surgery characteristics			
Surgery duration (min)	163.8 ± 22.0	162.8 ± 23.1	0.723
Surgery types			0.521
Wedge resection [*n* (%)]	8 (6%)	6 (4%)	
Segmentectomy [*n* (%)]	18 (13%)	22 (16%)	
Lobectomy [*n* (%)]	84 (61%)	92 (67%)	
Extended lobectomy [*n* (%)]	25 (18%)	16 (12%)	
Pneumonectomy [*n* (%)]	3 (2%)	2 (1%)	
Anesthesia characteristics			
Anesthesia duration (min)	175.0 ± 22.1	177.2 ± 22.3	0.413
OLV duration (min)	108.5 ± 15.5	108.6 ± 12.2	0.945
Extubation time (min)	38 (26–47)	34 (26–43)	0.097
Fluid management (ml)			
Total infusion volume	900 (800–1100)	700 (600–900)	< 0.001
Crystalloid infusion volume	500 (400–600)	500 (450–650)	0.066
Colloid infusion volume	400 (300–500)	150 (100–200)	< 0.001
Estimated blood loss	100 (60–165)	100 (50–150)	0.335
Urine volume	200 (120–300)	160 (110–240)	0.011
Catecholamines			
Norepinephrine (μg)	32 (16–48)	480 (360–640)	< 0.001
Dopamine (mg)	3 (1–6)	NA	
Phenylephrine (μg)	200 (100–400)	NA	
Postoperative characteristics			
First mobilization time (h)	10 (8–15)	12 (10–15)	0.066
ICU LOS (h)	8 (7–10)	9 (7–10)	0.475
Hospitalization LOS (d)	8 (6–10)	8 (7–10)	0.831

Continuous data are presented as mean ± SD or median [interquartile range (IQR)]; categorical data are presented as number (percentage)

*Abbreviations*: *OLV* One-lung ventilation, *ICU* Intensive care unit, *LOS* Length of hospital stay

### Postoperative complications and biochemical parameters

**Fig. 4:**
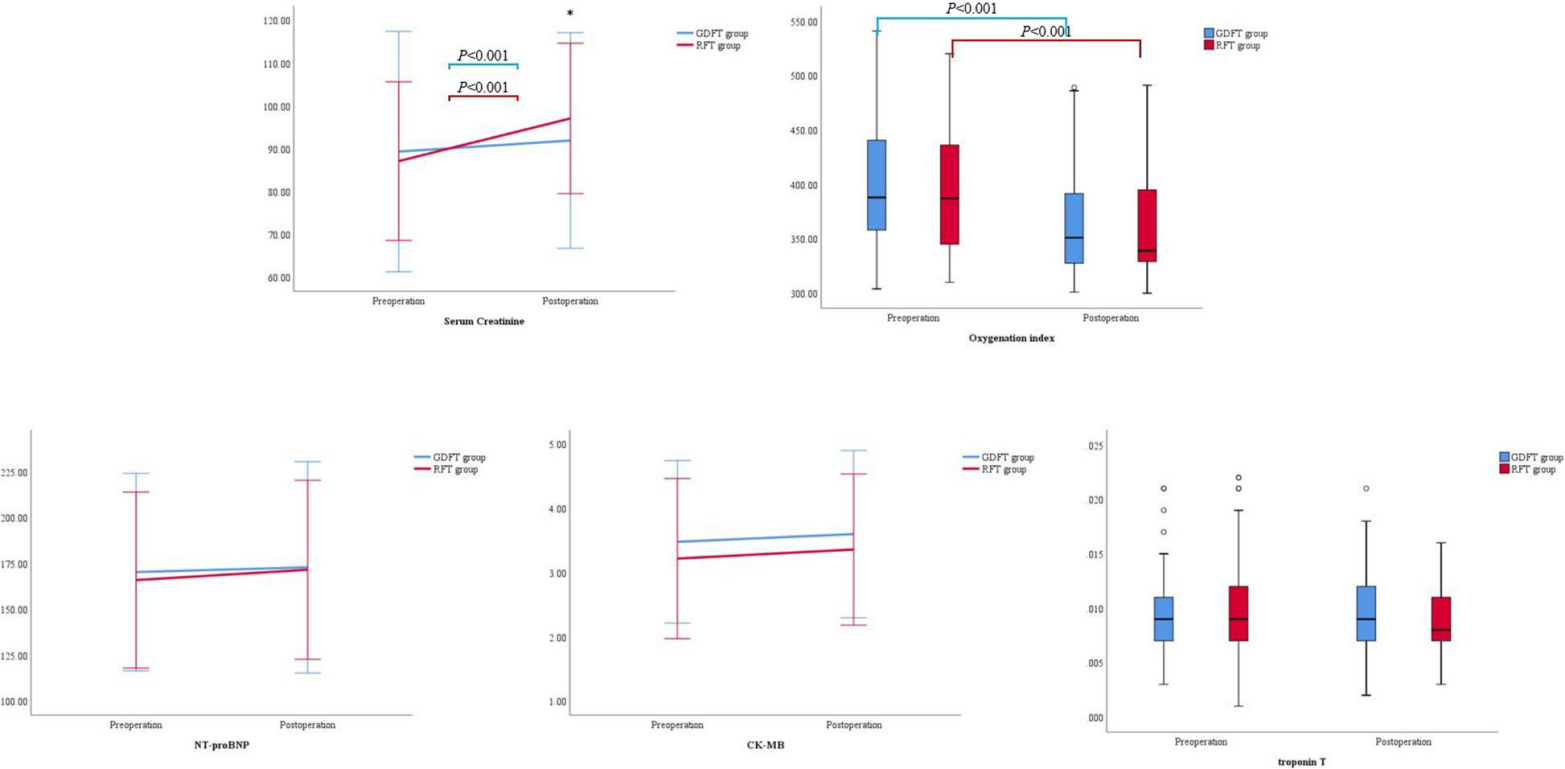
The preoperative and postoperative biochemical parameters. *Compared between groups, *P* = 0.048

There were no significant differences in the postoperative AKI, pulmonary, and cardiac complications between the two groups (Table 3). The serum creatinine was increased, and the oxygenation index was decreased significantly after operation in both groups. The postoperative serum creatinine was lower in GDFT group than that in RFT group (GDFT vs RFT; 91.9 ± 25.2 μmol/L vs 97.1 ± 17.6 μmol/L; *P* = 0.048); there was not significant difference of postoperative oxygenation index between the two groups. There were no significant differences in NT-proBNP, CK-MB, and troponin T before and after operation in two groups (Fig. 4).

**Table 3:** Postoperative complications

Parameters [*n* (%, 95% CI)]	GDFT group (*N* = 138)	RFT group (*N* = 138)	*P*-value
AKI	6 (4.3%, 0.9–7.8%)	11 (8.0%, 3.4–12.5%)	0.317
ALI	15 (10.9%, 5.6–15.1%)	17 (12.3%, 6.8–17.9%)	0.851
Pneumonia	14 (10.1%, 5–15.2%)	12 (8.7%, 3.9–13.5%)	0.837
MINS	8 (5.8%, 1.8–9.7%)	11 (8.0%, 3.4–12.5%)	0.636
Heart failure	2 (1.4%, 0.6–3.5%)	1 (0.7%, 0.7–2.2%)	1.000
New onset arrhythmia			
Tachycardia	7 (5.1%, 1.4–8.8%)	4 (2.9%, 0.1–5.7%)	0.540
Atrial fibrillation	4 (2.9%, 0.1–5.7%)	6 (4.3%, 0.9–7.8%)	0.749
Ventricular premature	10 (7.2%, 2.9–11.6%)	8 (5.8%, 1.8–9.7%)	0.808

Data are presented as number (percentage)

*Abbreviations*: *CI* Confidence interval, *AKI* Acute kidney injury, *ALI* Acute lung injury, *MINS* Myocardia injury after noncardiac surgery

### Intraoperative hemodynamic parameters

**Fig. 5:**
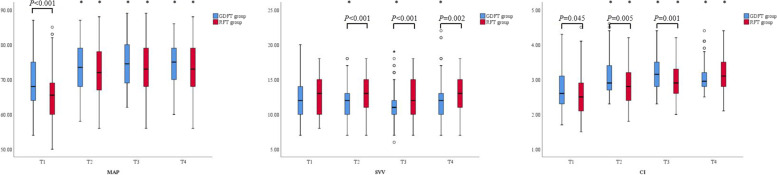
The intraoperative hemodynamic parameters. *MAP* mean arterial pressure, *SVV* stroke volume variation, *CI* cardiac index. *Compared to *T*_1_ within groups, *P* < 0.05

MAP, SVV, and CI were recorded at four time points: after anesthesia induction (*T*_1_), 10 min after OLV (*T*_2_), 10 min after lung recruitment (*T*_3_), and the end of operation (*T*_4_). MAP was higher at *T*_1_ in GDFT group than in RFT group; they were increased after induction, but there were no significant differences at *T*_2_, *T*_3_, and *T*_4_ between the two groups. SVV was decreased after induction in GDFT group; it was lower at *T*_2_, *T*_3_, and *T*_4_ in GDFT group than in RFT group. CI was increased after induction in two groups; it was higher at *T*_1_, *T*_2_, and *T*_3_ in GDFT group than in RFT group (Fig. 5).

## Discussion

AKI indicates a rapid decrease in renal function with a broad spectrum of severity, ranging from mild serum creatinine elevation to the complete loss of renal function. Postoperative AKI accounted for 18–47% of all causes of hospital-acquired AKI (Romagnoli and Ricci 2015) and associated with considerable morbidity and mortality (Gumbert et al. 2020). One study showed that the incidence of AKI was 1.8% in patients undergoing thoracoscopic lobectomy, but elder patients and patients with coronary artery disease (CAD) were excluded (Wu et al. 2019). Another study showed that the incidence of AKI was 6% after open lobectomy surgery (Kim et al. 2020). The incidence of AKI in our study was 6.2%. The patients in our study were elderly, most patients have smoking history, the comorbidities such as hypertension and diabetes mellitus were high, and the operation duration was more than 2 h; these were all risk factors of postoperative AKI (Löffel et al. 2020; Hallqvist et al. 2018), so the incidence of AKI was high in our study.

There were several diagnostic criteria of AKI (Zarbock et al. 2018); the diagnostic indicators included serum creatinine and urine output. The postoperative urine output record was inaccurate because the urinary catheter was removed immediately after anesthesia recovery in our study; studies also showed that urine output was unrelated to intraoperative infusion volume and perioperative kidney function (Matot et al. 2013; Hahn 2010), so only serum creatinine level was used to diagnosis AKI in our study. We also did not grade AKI according to the degree of serum creatinine increasing or the degree and duration of urine output decreasing in our study.

The relationship between fluid administration and kidney function has been studied. Compared to intraoperative infusion rate of 10.9 ml/kg/h, intraoperative infusion rate of 6.5 ml/kg/h was associated with a higher AKI incidence after major abdominal surgery (Myles et al. 2018). Another study of 92,094 patients undergoing noncardiac surgery showed an increased incidence of AKI when intraoperative fluid volume was less than 900 ml for a 3-h operation (Shin et al. 2018). In open thoracic surgery, compared to intraoperative net infusion rate of more than 6 ml/kg/h, the AKI incidence was higher when intraoperative net infusion rate is of less than 3 ml/kg/h (Kim et al. 2020). In our study, the total infusion volume was higher in GDFT group than that in RFT group, though there was no significant difference of AKI incidence between the two groups, but the postoperative increase degree of serum creatinine was lower in GDFT group than that in RFT group. The benefit may from the higher infusion volume and lower dosage of norepinephrine usage and the use of dopamine in GDFT group.

GDFT using fluids, inotropes, and vasopressors, but not fluids alone, could reduce the postoperative complications, because the bundle protocol could improve hemodynamics and tissue perfusion (Dushianthan et al. 2020). Compared to intraoperative infusion rate of 6.5 ml/kg/h, patients who received infusion rate of 10.9 ml/kg/h during major abdominal surgery had higher CI at the end of operation, because the higher CI could increase perfusion in organs sensitive to hypovolemia; this may explain the lower rate of AKI in the liberal fluid group (Phan et al. 2021). In our study, intraoperative CI were higher in GDFT group than that in RFT group; the higher CI may be another explanation of lower increase degree of postoperative serum creatinine in GDFT group.

Both colloids and crystalloids can be used in GDFT protocol. Meta-analysis showed that postoperative kidney dysfunction was similar between colloid-based GDFT and crystalloid-based GDFT after major noncardiac surgery (Tyagi et al. 2020). In our study, HES was used for fluid bolus in GDFT protocol. Though HES has adverse effect in severe sepsis patients and intensive care patients (Perner et al. 2012; Myburgh et al. 2012), but the pathophysiology was different between sepsis and operation patients. A study of 11,691 patients who underwent elective noncardiac surgeries showed that the intraoperative use of moderate doses of 6% HES 130/0.4 was not associated with increased risk of AKI (Degoul et al. 2020), as while as in cardiac surgery (Nagore et al. 2021) and liver transplantation surgery (Chen et al. 2021). The recent meta-analysis also showed that HES was shown to be safe and efficacious in the perioperative setting (Chappell et al. 2021). In thoracic surgery, AKI occurred more often only when HES was administered to the patients with decreased renal function or having more than two risk factors with normal renal function. The risk factors included the following: angiotensin-converting enzyme inhibitor/angiotensin receptor blockers, open thoracotomy, pneumonectomy/esophagectomy, diabetes mellitus, cerebrovascular disease, and low albumin level (Ahn et al. 2016). There were few patients who met these standards in our study; the use of HES has not actually offset the benefit of GDFT.

Meanwhile, colloids were more effective at expanding blood volume and stabilizing hemodynamic parameters (Martin and Bassett 2019), thus avoiding excessive fluid infusion. Excessive perioperative fluid administration was a risk factor of PPCs. Study showed that intraoperative fluid management more than 6 ml/kg/h was independent risk factor of PPCs after thoracoscopic lung resection (Kaufmann et al. 2019); the PPCs were very high after lung resections when intraoperative infusion rate was more than 8 ml/kg/h (Arslantas et al. 2015). In our study, the infusion rate was less than 6 ml/kg/h in both groups; the incidence of PPCs was low and consistent between the two groups. The cardiac complications after pulmonary lobectomy were low and more related to surgical trauma (Sanaiha et al. 2019), so there were no significant differences of cardiac complications between the two groups.

Our study also has limitations. First, the definition of high-risk patients was not accurate enough, risk scores were not calculated, and this may be a potential confounder. Second, fluid management after operation was not standardized, it may skew the effect of intraoperative fluid optimization. Third, the implementation rate of some ERAS program items was low; it may affect the results. Fourth, grading AKI and comparing the incidence of AKI of different stages may be more meaningful. Unfortunately, we did not grade AKI in our study.

## Conclusions

Under ERAS program, there was no significant difference of AKI incidence between GDFT and RFT in elderly patients undergoing thoracoscopic pulmonary resection. But postoperative increase degree of serum creatinine was lower in GDFT group.

## Data Availability

The datasets used and analyzed during the current study are available from the corresponding author on reasonable request.

## Abbreviations

AKI: Acute kidney injury
LOS: Hospitalization length of stay
ERAS: Enhanced recovery after surgery
ICU: Intensive care unit
RFT: Restrictive fluid therapy
GDFT: Goal-directed fluid therapy
PPCs: Postoperative pulmonary complications
BUN: Blood urea nitrogen
NT-proBNP: N-terminal pro-B-type natriuretic peptide
HES: Hydroxyethyl starch
OLV: One-lung ventilation
SVV: Stroke volume variation
CI: Cardiac index
MAP: Mean arterial pressure
ALI: Acute lung injury
MINS: Myocardia injury after noncardiac surgery
CK-MB: Creatine kinase-MB
IQR: Interquartile range
CAD: Coronary artery disease
